# Multisystem Inflammatory Syndrome With Complete Kawasaki Disease Features Associated With SARS-CoV-2 Infection in a Young Adult. A Case Report

**DOI:** 10.3389/fmed.2020.00428

**Published:** 2020-07-14

**Authors:** Elie Cogan, Pierre Foulon, Olivier Cappeliez, Nicole Dolle, Gaëlle Vanfraechem, Daniel De Backer

**Affiliations:** ^1^Department of Internal Medicine, CHIREC Hospital, Brussels, Belgium; ^2^Université Libre de Bruxelles (ULB), Brussels, Belgium; ^3^Department of Intensive Care, CHIREC Hospital, Brussels, Belgium; ^4^Department of Radiology, CHIREC Hospital, Brussels, Belgium; ^5^Department of Biochemistry, CHIREC Hospital, Brussels, Belgium; ^6^Department of Infectious Diseases, CHIREC Hospital, Brussels, Belgium

**Keywords:** case report, Kawasaki disease, COVID-19, SARS-CoV-2, MIS-C, tocilizumab, serology

## Abstract

A severe multisystem inflammatory syndrome associated with Kawasaki disease manifestations (MIS-C) has been recently reported in children with signs of recent infection with SARS-CoV-2. We here reported the case of a young adult woman who presented the complete manifestations of Kawasaki disease associated with a severe myocarditis, acute respiratory distress syndrome and hemodynamic instability a few weeks after a transient anosmia. The detection of specific antibodies to SARS-CoV-2 in the absence of detection of the virus suggested that the syndrome was the result of a delayed immune response to a recent COVID-19 infection. A combined treatment with colchicine, tocilizumab, high dose immunoglobulins, and methylprednisolone allowed to control the inflammatory process and to limit the development of coronary aneurysm. The patient recovered without sequelae. This case emphasized the importance of SARS-CoV-2 serology for the diagnosis of delayed immune complications of COVID-19. Clinicians caring for adult patients must be aware that not only children but also young adults can be affected by a multisystem inflammatory syndrome with KD features associated with COVID-19.

## Introduction

Kawasaki's disease (KD) is a rare acute febrile disease affecting mostly children characterized by the association of conjunctivitis, erythema of the lips and oral mucosa, polymorphous exanthema, palmar-plantar erythema, and cervical lymphadenopathy. KD is a widespread vasculitis affecting small and medium sized arteries, with the possible occurrence of coronary aneurysms (1). Lung involvement is exceptional in KD.

Recently, Riphagen et al. (2) reported that previously healthy children presented a hyperinflammatory shock with Kawasaki disease-like features in association with infection with severe acute respiratory syndrome coronavirus 2 (SAR-CoV-2). Shortly after, Verdoni et al. (3) confirmed these data, reporting that the incidence of KD was much higher in their area during the SARS-CoV-2 outbreak that in the same period in preceding years. This led Center of Disease Control (CDC) to publish an alert (HAN00432) on May 14, warning physicians on the occurrence of a multisystem Inflammatory Syndrome in Children (acronym “MIS-C”) associated with SAR-CoV-2 (4). The case definition of MIS-C as defined by the CDC concerned an individual aged <21 years presenting fever >38°C for ≥24 h, laboratory evidence of inflammation and evidence of clinically severe illness requiring hospitalization, with multisystem (≥2) organ involvement (cardiac, renal, respiratory, hematologic, gastrointestinal, dermatologic, or neurological) and no alternative plausible diagnoses and positive for current or recent SARS-CoV-2 infection (4). Interestingly, even though CDC asked to report cases of patients younger than 21 years old, no case older than 15 years has yet been published.

We report the delayed occurrence of a multisystem inflammatory syndrome with complete Kawasaki disease features in a young adult patient recently infected by SARS-CoV-2.

## Case Presentation

A 19.9 year-old woman of Caucasian origin without any significant personal or familial past history presented a sudden transient loss of smell on March 25, 2020 without any additional symptoms. She had participated without wearing a mask in a yoga session with several other people on March 12 and had been confined at home since March 13. Her parents were the only daily contacts. Between March 13 and March 21, her mother has been in close professional contact with a sick colleague which was finally diagnosed as severe Covid-19, but serologic tests performed end April in the mother and father were negative.

On April 14, she developed a febrile illness associated with cervical adenopathy, a morbilliform erythematous rash affecting the forearms, the hands and the buttocks, red and edematous lips and bilateral conjunctivitis with palpebral edema.

**Figure 1:**
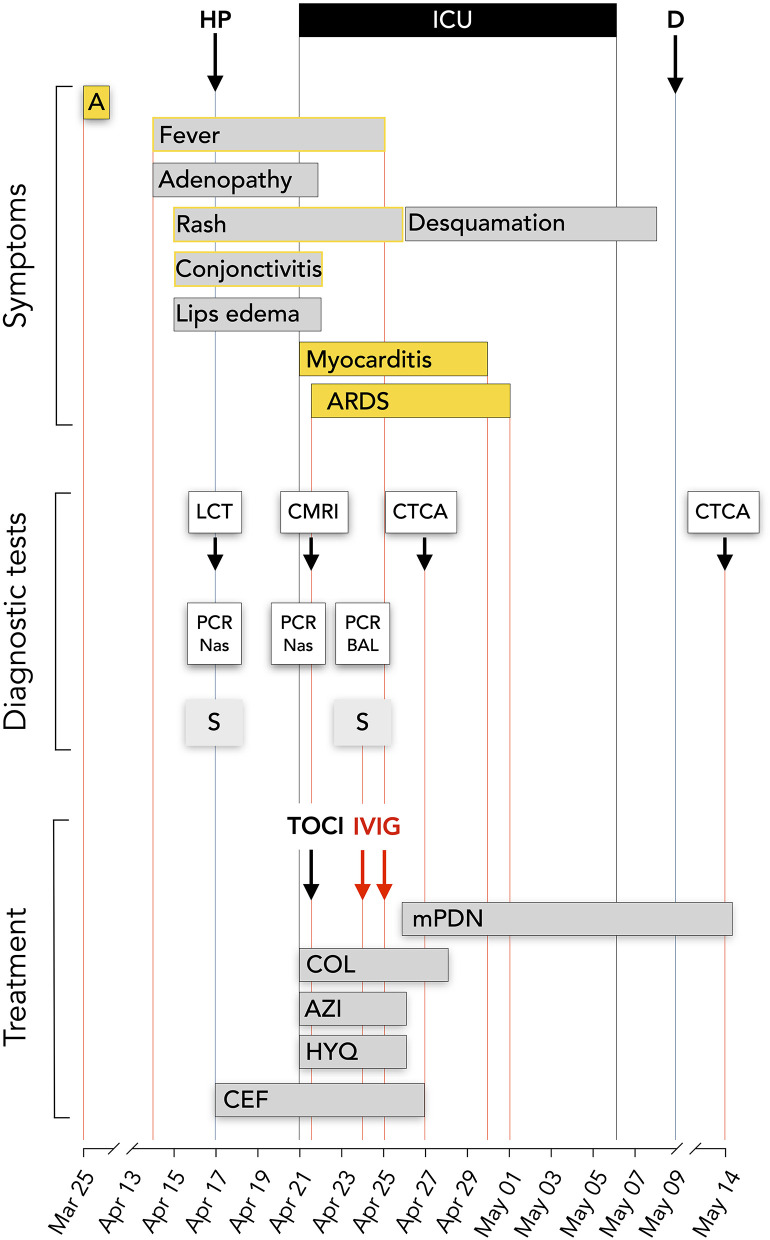
Time line. Symptoms, diagnostic tests and treatment. HP, hospitalization; ICU, intensive care unit; D, discharged day; A, transient anosmia; LCT, lung computed tomography; CMRI, cardiac magnetic resonance imaging; CTCA, computed tomography coronary angiography; PCR Nas, SAR-CoV-2 PCR on nasopharyngeal smear; PCR BAL, SAR-CoV-2 PCR on bronchoalveolar lavage; S, SAR-CoV-2 serology; HYQ, Hydroxychloroquine 400 mg bid during 2 days and then 200 mg; AZI, Azithromycine 500 mg on day 1 than 250 mg/day; COL, Colchicine 0.5 mg bid; mPDN, methyprednisolone 60 mg IV bid initially, 48 mg oral dose at discharge; 24 mg at May 14; CEF, ceftriaxone 2 g/d; TOCI, tocilizumab IV 480 mg; IVIG, Privigen 60 g.

**Figure 2:**
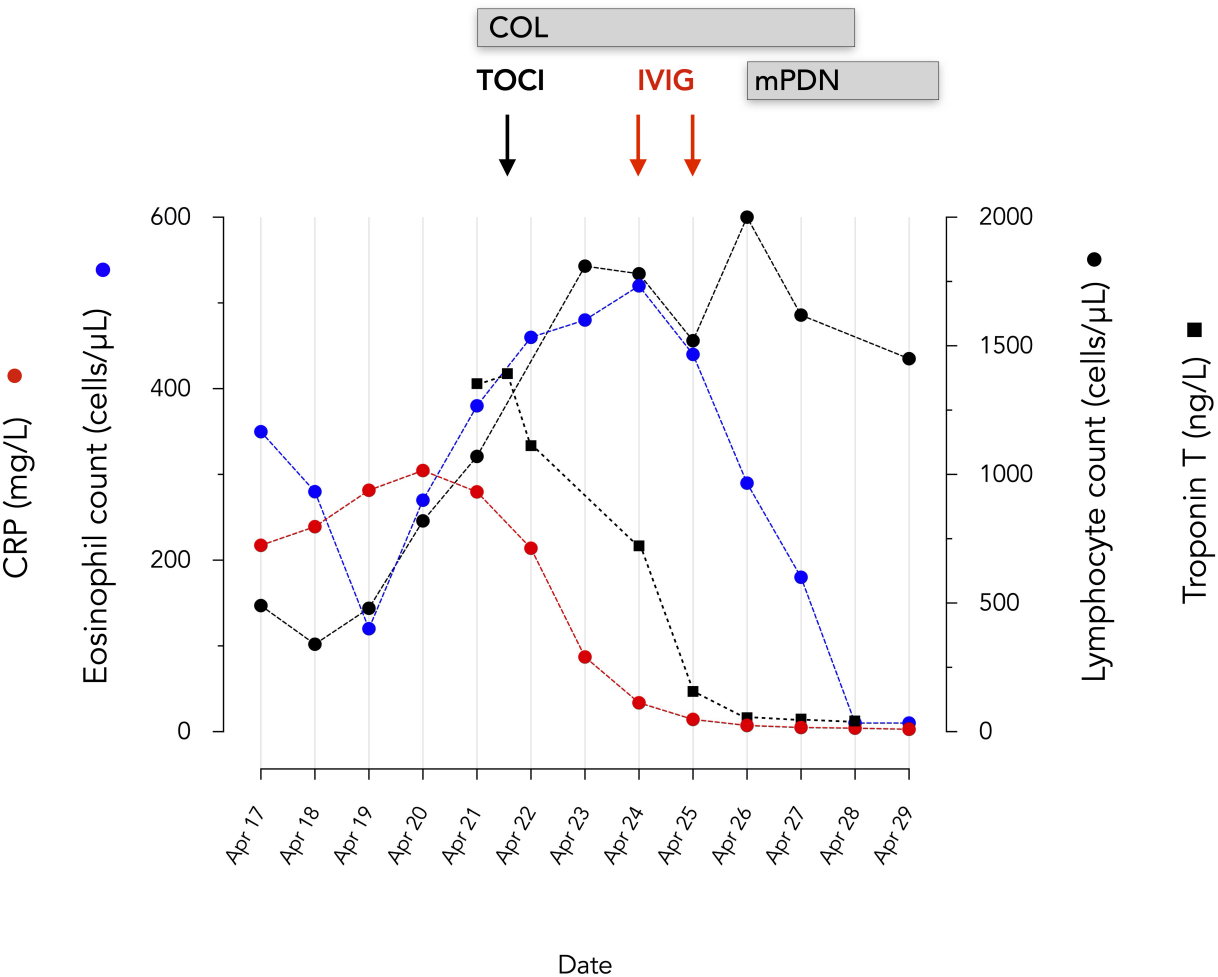
Evolution of eosinophils, lymphocytes, serum CRP and troponin T. mPDN, methyprednisolone 60 mg IV bid initially, 48 mg oral dose at discharge; 24 mg at May 14; COL, Colchicine 0.5 mg bid; TOCI, tocilizumab IV 480 mg; IVIG, Privigen 60 g.

**Figure 3:**
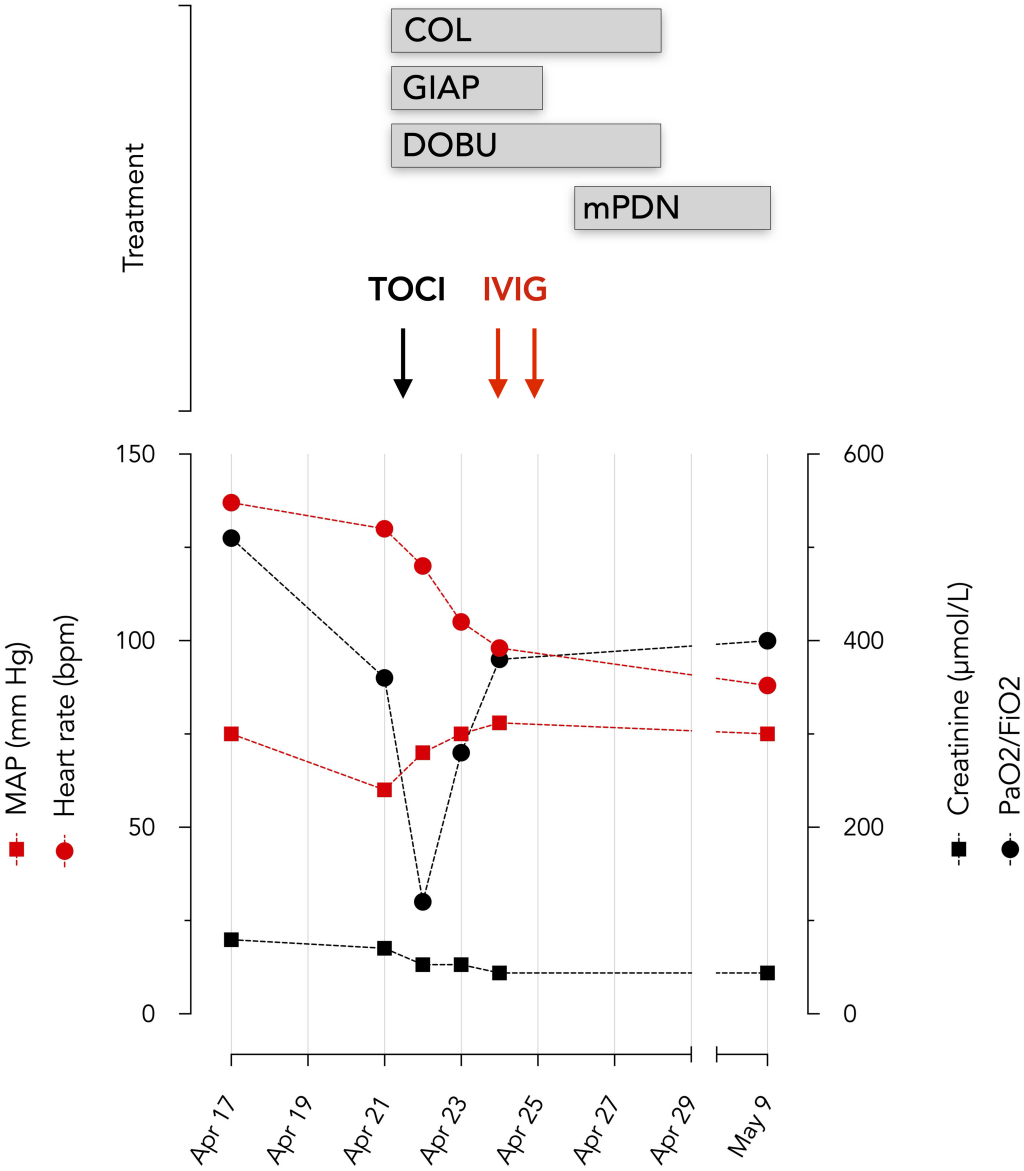
Evolution of hemodynamic and organ function variables. Oxygenation was assessed by PaO_2_/FiO_2_ ratio and renal function by serum creatinine levels BPM beats per minute. TOCI, tocilizumab; COL, colchicine; DOBU, dobutamine; mPDN, methylprednisolone; IVIG, Privigen 60 g; GIAP, Giapreza.

On April 17, she was admitted at the CHIREC hospital. On hospital admission, the heart rate was 137/min and the arterial blood pressure 129/73 mm Hg; she was not overweight (weight: 60 kg; BMI 24.7 kg/m^2^); the throat was red, the cervical adenopathy was enlarged and painful; conjunctivitis and skin lesions were still present; lungs auscultation was clear and excepting marked tachycardia, heart exam was normal. Between April 17 and April 21, the patient remained febrile with a persistent inappropriate tachycardia. Symptoms time line are depicted on Figure 1. On admission the main blood laboratory results were as follows: white blood cells 11,100/μL, neutrophils 9,730/μL, lymphocytes 490/μL, eosinophils 350 /μL, platelets 147,000/μL, CRP 217 mg/L, fibrinogen 759 mg/dL, ferritin 285 μg/L, Na 131 mmol/L, creatinine 79,4 μmol/L, ASAT 52 U/L (*n* < 32). The evolution of principal blood parameters is depicted in Figure 2 and hemodynamic and organ function variables in Figure 3.

A transthoracic echocardiography (TTE) demonstrated a severely decreased ejection fraction of the left ventricle (LVEF 15%), hyperechoic aspect of pericardium and small posterior pericardial effusion associated to a marked increased serum troponin T. A cardiac magnetic resonance imaging demonstrated myocardial edema typical of acute myocarditis. The mean arterial blood pressure dropped to 60 mm Hg with decreased oxygenation conditions and the patient was transferred to the ICU.

The first hemodynamic profile demonstrated a mean arterial pressure at 60 mmHg, a cardiac index at 2.1 l/min.M^2^ and a central venous O_2_ saturation (ScvO_2_) at 47%. Dobutamine was initiated and an invasive hemodynamic monitoring device (PiCCO 5F catheter, Gettinge, Germany) was inserted. The invasive hemodynamic assessment under 5 mcg/kg min of dobutamine, reported a cardiac index at 3.7 L/min M^2^ (normal 2.5–3.5), systemic vascular resistance at 685 dyne s cm^−5^ (normal 800–1,200), a global end diastolic volume at 932 ml/M^2^ (normal 600–800), an extravascular lung water index at 17 ml/kg (normal <10) and a ScvO_2_ at 63%, which suggests distributive shock with marked myocardial depression. Given this hemodynamic profile, inotropic, and vasopressor support was required for several days.

Dobutamine was infused from April 21 to April 28 (maximum dose 5 μg/kg/min) and synthetic human angiotensin 2 (Giapreza, maximum dose 20 mg/kg/min) from April 21 to April 25.

Multiple attempts of weaning these agents were performed daily. Despite hemodynamic stabilization, she rapidly developed ARDS according to Berlin criteria (5). She was mechanically ventilated and proned. Extensive workout was performed to rule out ongoing infections (including a bronchoalveolar lavage which disclosed an inflammatory pattern with predominance of neutrophils, without any detectable strain at direct examination, culture, as well as PCR for multiple respiratory pathogens). A gynecologic examination and multiple bacterial samplings were negative. There were no signs of macrophage activating syndrome (normal triglycerides—normal LDH- absence of very elevated ferritin). Serum IL-6 was 306 ng/mL (*N* < 20) and D-Dimer progressively increased from 3.9 μg/ml (*N* < 0.5) to 17.8 μg/ml.

The diagnosis of SARS-CoV-2 infection was considered. PCR tests were negative on two nasopharyngeal smears and on bronchoalveolar lavage but IgG and IgM against SARS-CoV-2 were detected on a blood sample taken on admission by the rapid test (Zhejiang Orient Gene Biotech Co., Ltd). A quantitative Ig G determination by chemiluminescence technology (DiaSorin, Italy) demonstrated an increase in specific IgG antibodies from 13.7 U on admission to 25 U after 7 days (negative <12 arbitrary units; positive >15 arbitrary units).

Given the consideration of SARS-CoV-2 related ARDS, myocarditis and distributive shock, tocilizumab (RoActemra, Roche), 480 mg was infused. PaO_2_/FiO_2_, CRP, and Troponin T rapidly improved (Figures 2, 3). Impairment in myocardial function was resolved within 48 h.

**Figure 4:**
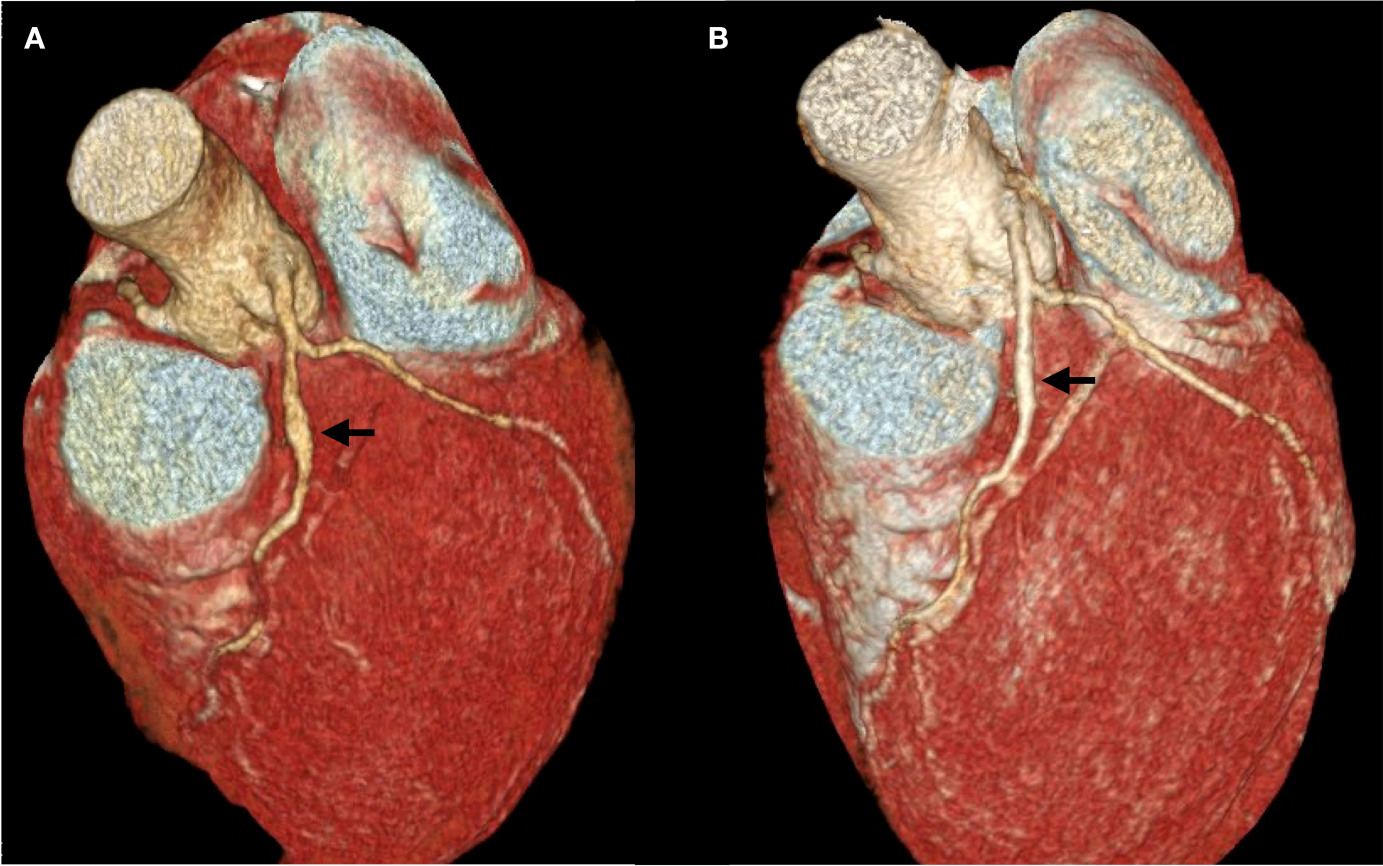
Computed Tomography Coronary Angiography. **(A)** The first exam was performed on April 27, 13 days after the onset of the disease, 6 days after tocilizumab administration and 3 days after intravenous immunoglobulins. A 5 mm fusiform ectasia is present at the proximal part of the anterior interventricular coronary artery (arrow). The external diameter of the artery is 3 mm above and below the ectasia; dilation area 19,3 mm^2^. **(B)** Second exam, performed on May 14, demonstrating a marked decrease in aneurism size. Four millimeter fusiform ectasia; dilation area 10,6 mm^2^.

Two days later, KD was considered based on clinical signs and significant eosinophilia; 1 g/kg intravenous immunoglobulins (Privigen, CSL Behring) were administered. A computed tomography coronary angiography (CTCA) demonstrated a coronary artery aneurism and high dose steroids were initiated, resulting in significative improvement 17 days later (Figure 4).

The patient fully recovered and was discharged on May 9.

## Discussion

This patient fulfilled all the criteria of typical KD (1) in addition to severe ARDS, distributive shock and severe myocarditis in the context of a recent SARS-CoV-2 infection suggested by the detection of IgG and IgM against SARS-CoV-2. She also fulfilled the criteria of the novel multisystem inflammatory syndrome reported in children being infected by SARS-CoV-2 (4).

The association of KD and multisystem inflammation with SARS-CoV-2 in children (MIS-C) has been reported in several European countries (2, 3, 6–10) and in US (11–13). The syndrome has been more recently requalified as pediatric inflammatory multisystem syndrome (PIMS) (6, 10, 14). As of the 15 of May 2020, in total, about 230 suspected cases have been identified in EU/EEA countries and the UK, including two fatalities (15). In France, respectively, 79 confirmed and 29 probable/possible PIMS related to SARS-CoV-2 (CoV-PIMS) have been collected between March 1 and May 17 (6). Of interest, the peak of the epidemic curve of PIMS occurred 4–5 weeks after the peak of the COVID-19 epidemic suggesting a delayed immune response to the virus. In Belgium, following the initial alerts for a Kawasaki-like disease mid-April 2020, a specific survey was set up by a pediatrician COVID-19 task force allowing centralized voluntary-based reporting of KD like/PIMS-TS by pediatricians across de country. Approximatively 25 cases of PIMS-TS have been reported through this survey up until June 23 (personal communication: Pr Stéphane Moniotte; Department of pediatrics, Cliniques universitaires Saint-Luc, UCLouvain, Brussels).

Of interest, in the largest published series, the median age was 8 years and 96% of the children were under the age of 16. No case older than 18 was reported yet (6).

There is no equivalent registry for adult patients and our case is the first to be reported so far. By the way it is important to acknowledge that the first report of KD or PIMS were published online on April 27, several days after the onset of the disease in this patient.

Lung involvement and ARDS are extremely rare in classical KD unrelated to COVID-19 while these are common in SARS-CoV-2 infection as well as in CoV-PIMS. Indeed, critical care support was required in two third of CoV-PIMS cases, 43% requiring mechanical ventilation (6). On the other hand, infection with SAR-CoV-2 is associated with an uncontrolled inflammatory response and widespread endothelial cell dysfunction (16). As such, KD and SAR-CoV-2 share some similar pathophysiological mechanisms, which can lead to MIS-C/PIMS.

Admittedly, an important question is whether KD results from a late response to a recent infection or whether the infection was ongoing. On the one hand, the absence of detectable virus in the respiratory tract is in accordance of a late immune complication of viral infection in the absence of residual virus. The precise dates of contamination and of acute infection with SARS-Cov-2 remain difficult to determine with precision in our patient. The contamination could have occurred before March 13, the time of confinement. Alternatively, it is not excluded that our patient could have been contaminated by her mother during the confinement period but the negative serology performed in late April in the mother does not confirm this possibility. However, we cannot totally exclude that possibility since it appears that IgG antibodies against SARS-CoV-2 might disappear rapidly particularly in asymptomatic individuals (17). Finally, given the occurrence of an anosmia in our patient, even of a short duration, we considered the date of March 23 as that of a possible paucisymptomatic infection.

Such hypothesis in consistent with a 3 weeks delay between the infection and the onset of KD as reported in several other similar cases (6).

On the other hand, an ongoing infection may not be excluded given the rise in IgG during hospital admission and the lack of sensitivity of the PCR. Importantly, the pediatric literature has also reported that a minority of patients had positive RT-PCR nasal swabs while most were diagnosed positive for SARS-CoV-2 by serology. Hence, it is likely that SARS-CoV-2 infection triggers the massive cytokine storm which is responsible for the KD symptoms, ARDS and myocarditis. This immune response is likely to be delayed emphasizing the importance of performing SARS-CoV-2 serologic tests in these patients with diagnostic uncertainty.

We suggest that even in asymptomatic or paucisymptomatic individuals the SARS-CoV-2 infection hits the respiratory tract which became more sensitive to the immune consequences associated to KD. Old studies have suggested that the viral infection may play a role as a superantigen that drives an autoimmune response via clonal expansion of CD8 T cells (18). The theory of T cell activation by a superantigen that could be instrumental in KD was also suggested by Brogan et al. (19). More recently the discovery of the presence of IgA plasma cells together with an oligoclonal IgA response in arterial tissues from patients with KD suggest that the immune response is driven by entry of a pathogen at a mucosal site such as the respiratory tract (20). Ig A plasma cells infiltration was also identified in the proximal respiratory tract in acute KD (21). It is noteworthy that infection with SARS-CoV-2 is linked to the presence of the ACE2 and TMPRSS2 receptors in the same tissues (22).

Genetic susceptibility to abnormal immune responses to infectious agents play key roles in initiating KD. Th 17 expansion and Treg depletion could be the hallmarks of acute KD (23). Indeed, an imbalance between T helper 17 lymphocytes and regulatory T cells with very increased inflammatory cytokines in the acute phase of KD is suggested by some studies (24). Interestingly, the same kind of Th17 type response that contributes to the cytokine storm has been demonstrated to be involved in pulmonary viral infections including SARS-CoV-2 (25).

All these data are consistent with the hypothesis that KD is a consequence of an immune mediated endothelial cell damage likely triggered by an acute viral infection affecting the respiratory system. Otherwise, an association between another coronavirus (HCoV-NH) and KD has been previously reported (26). Recent studies reported an increased incidence of KD associated to SARS-CoV-2 infection in children (2, 3). The hypothesis of a delayed onset of KD rather than an association with acute SARS-CoV-2 infection is suggested by the lack of detection of the virus in most affected children and the increased incidence of KD at time when the number of new COVID-19 cases decreases. However, in some other cases both diseases could be contemporary (3). In the present case, the occurrence of the inflammatory disorder 3 weeks after a possible pauci symptomatic SARS-CoV-2 infection (transient anosmia in the absence of other symptoms) favored the hypothesis of a delayed immune mechanisms induced by the viral infection as proposed by Belot et al. (6).

Tocilizumab, an anti-IL-6 receptor monoclonal antibody has been reported to be effective for patients with severe COVID-19 pneumonia and hyperinflammatory syndrome (27). It is also effective for the inflammatory syndrome associated to Kawasaki's disease in children (28). However, tocilizumab may have contributed to the formation of new-onset of coronary artery aneurysms in these children (28). As KD was not yet recognized at time of acumen of ARDS, myocardial dysfunction and distributive shock, we administered tocilizumab for its potential beneficial effects in this setting. We here noticed a very positive response to tocilizumab on ARDS and signs of cardiac involvement (troponin and cardiac function at echocardiography) which rapidly improved after tocilizumab administration. However, a cardiac aneurism was detected at CTCA 3 days later. While the development of cardiac aneurisms after tocilizumab therapy in KD is consistent with the previous observations of Nozawa et al. (28), it is important to notice that the onset of aneurism in the KD children treated with tocilizumab was quite late (several weeks), and it is thus possible that the aneurism pre-existed tocilizumab therapy in this patient. The regression of the coronary aneurysm after IVIG and methylprednisolone administration may suggest that these treatments are a mandatory part of the therapy when cardiac aneurisms are present, particularly for patients treated by tocilizumab. Apart from the administration of tocilizumab, the initiation of corticosteroid therapy was justified by the risk of non-response to IVIG. Indeed, the Kobayashi score had been evaluated at 6 points which constitutes a 50% risk of IVIG ineffectiveness in KD (29). The benefit of prednisolone administration is well-documented in these circumstances (30, 31).

It is interesting to notice that eosinophils, which have been associated with the development of more severe coronary vasculitis in KD (32) continued to increase despite tocilizumab even though troponin and CRP decreased. The eosinophilia detected in our patient with KD contrasts with the marked eosinopenia characteristic of patients with severe forms of COVID-19 (33, 34). Although KD is associated with a Th17 rather than a Th2 immune response, the pathogenic role of eosinophils in KD is underlined by their presence in the inflammatory infiltrates characterizing the coronary vasculitis of KD (20, 32). In addition, eosinophilia, sometimes marked, was present in all patients with KD developing coronary aneurysms (32). In the context of a COVID-19 epidemic, a blood eosinophilia could be a useful tool raising suspicion of KD.

Additionally, eosinophils may be an important indicator for requirement of additional glucocorticoid therapy in patients with multisystem inflammatory syndrome associated with SARS-CoV-2, especially when other markers of inflammation seem to be controlled.

There are several limitations to this report. First, KD was recognized relatively late in the course of the patient. It is important to acknowledge that the first report of KD or PIMS was published online on April 27, several days after the onset of the disease in this patient. This may have contributed to the late recognition of KD/PIMS in this patient. Also, several clinical features are common in KD and in acute SARS-CoV-2 disease: conjunctivitis, fever, rash, myocarditis. Accordingly, the initial diagnosis that was considered in this patient was SARS-CoV-2 ARDS and myocarditis, and KD was considered later in view of the continuous growth of eosinophils despite significant improvement of the other signs. Second, several drugs (hydroxychloroquine, azithromycin, colchicine) seem not indicated in KD. Again, these were indicated at time of consideration of SARS-CoV-2 ARDS, myocarditis and distributive shock. Colchicine was administered for myopericardial involvement. Of note colchicine has marked rheologic and anti-inflammatory properties, inhibits T-cell activation (35). It is a class IIa recommendation in pericarditis (36), and can even be used after acute myocardial infarction (37). Admittedly, its use in myocarditis has not been well-described, even though anti-inflammatory agents may have beneficial effects (38). Recently, Deftereos et al. (39) reported in a randomized study that colchicine improved the time to deterioration in patients hospitalized with SARS-CoV-2. Hydroxychloroquine and azithromycin were administered for SARS-CoV-2 ARDS. Ceftriaxone was administered in the context of ARDS and distributive shock, while waiting for the results of bacteriological sampling.

A final consideration regards the use of exogenous angiotensin 2 (Giapreza). After correction of the severe myocardial dysfunction with dobutamine, it became evident from the hemodynamic profile that the patient suffered from distributive shock. The choice of the vasopressor agent in the context of SARS-CoV-2 associated vasoplegia is still a matter of debate. Some theoretical considerations suggest that AT2 may have beneficial effects as exogenous AT2 administration is associated with internalization of AT2 receptors, a key receptor involved in the pathogenicity of SARS-CoV-2 (40). These theoretical considerations were supported by a case series by Zangrillo et al. (41) which demonstrated that AT2 administration was associated with a rapid improvement in gas exchanges and respiratory function.

## Conclusion

Clinicians caring for adult patients must be aware that not only children but also young adults can be affected by a multisystem inflammatory syndrome with KD features associated with COVID-19.

A careful clinical history is necessary to identify subtle symptoms (as loss of taste or smell) suggestive of SARS-CoV-2 infection in the preceding weeks as the symptoms of MIS associated with SARS-CoV-2 may mimic an acute onset of SARS-CoV-2.

As it appears to be a delayed immune reaction to SARS-CoV-2 infection, serology constitutes a mandatory diagnostic tool.

This case also suggests that co-administration of colchicine, tocilizumab, IVIG and corticosteroids had favorable effects on systemic inflammation and cardiac and pulmonary manifestations and may control the development of arterial coronary aneurysm.

## Data Availability Statement

All datasets presented in this study are included in the article/supplementary material.

## Ethics Statement

Written informed consent was obtained from the individual(s), and/or minor(s)' legal guardian/next of kin, for the publication of any potentially identifiable images or data included in this article.

## Author Contributions

EC and DD analyzed the data and wrote the paper. PF and GV take care of the patient. OC performed cardiac magnetic resonance imaging and CTCA. ND performed the serologic tests. All authors approved the final work.

## Conflict of Interest

The authors declare that the research was conducted in the absence of any commercial or financial relationships that could be construed as a potential conflict of interest.
